# Supplementary material for: A functional trade-off between trophic adaptation and parental care predicts sexual dimorphism in cichlid fish
Source: Proc Biol Sci. 2019 Aug 21;286(1909):20191050. doi: 10.1098/rspb.2019.1050 (PMC6732390; doi:10.1098/rspb.2019.1050)
Supplement: Supplementary Information [file rspb20191050supp1.pdf]

Supplementary Information

A functional trade-off between trophic adaptation and parental care predicts sexual dimorphism in cichlid fish

Fabrizia Ronco[1*], Marius Roesti[1,2*] & Walter Salzburger[1*]

[1] Zoological Institute, University of Basel, Vesalgasse 1, 4051 Basel, Switzerland
[2] Department of Zoology, University of British Columbia, 6270 University Boulevard, Vancouver, BC V6T1Z4, Canada
[3] Current address: Institute of Ecology and Evolution, University of Bern, 3012, Switzerland
* All authors contributed equally to this work

Authors for correspondence:
Fabrizia Ronco
e-mail: fabrizia.ronco@unibas.ch
Marius Roesti
e-mail: marius.roesti@iee.unibe.ch
Walter Salzburger
e-mail: walter.salzburger@unibas.ch

Sampling procedure

Fish were caught using gill nets while snorkeling or scuba diving, or bought from local fishermen. After euthanasia with clove oil, specimens were measured (standard length = SL) and the sex was determined whenever possible. For subsequent morphological measurements, the entire gill apparatus was extracted and stored in 96% EtOH. For the stable isotope analysis, entire specimens were fixed in 10% formalin for 4 days, rinsed with water and transferred to 70% EtOH.

CT-scanning (figure 2*b*)

The mouthbrooding female (*Paracyprichromis* sp.) was euthanised on ice, fixed in 10% formalin and then gradually transferred to 100% EtOH. To increase contrast of the surface of the developing eggs in the buccal cavity, the mouth was rinsed repeatedly with 5% Lugol's iodine (I3K). CT-scanning of the head region was carried out on a Bruker Skyscan 1174v2, at 50kV, 800μA using a 0.25mm Aluminium filter and 4500ms exposure time. Voxel size was 29.8μm with 600 projections. Reconstruction was performed using NRecon (Version: 1.6.10.2), post-processing and visualisation was done in CTvox (Version: 3.3). Eggs and gill rakers were afterwards highlighted on the image using Adobe Photoshop (CC 2017).

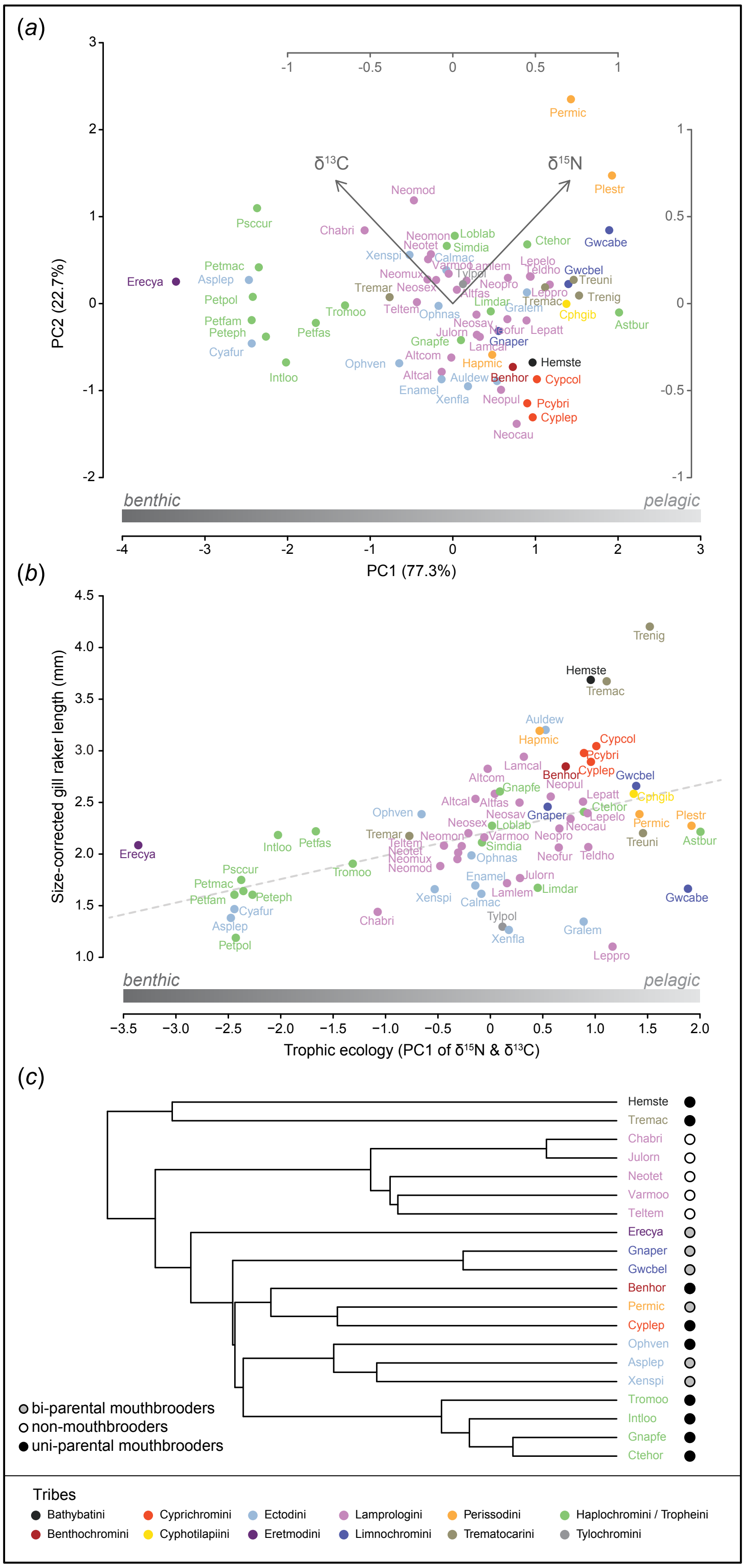

Figure S1: Trophic ecology of 65 Tanganyikan cichlid species, its correlation with gill raker morphology, and the distribution of the different breeding modes across the phylogeny (See supplementary table S1 for full species names): *(a)* A scaled Principal Component Analysis (PCA) of stable isotope measurements ($\delta^{15}N$ and $\delta^{13}C$, species means) was used to infer the major axis of variation across two major components of aquatic ecology: the benthic-pelagic ($\delta^{13}C$) and trophic ($\delta^{15}N$) position. We used PC1-scores (equally loaded with two components ($\delta^{15}N$: 0.71, $\delta^{13}C$: -0.71)) in downstream analyses as a univariate proxy for trophic ecology. ***(b)*** Phenotype-environment correlation between gill raker length and trophic ecology across 65 Tanganyikan cichlid species. Gill raker length (species mean) is positively associated with PC1-scores of stable isotope data. ***(c)*** The phylogenetic structure of the three different breeding modes.

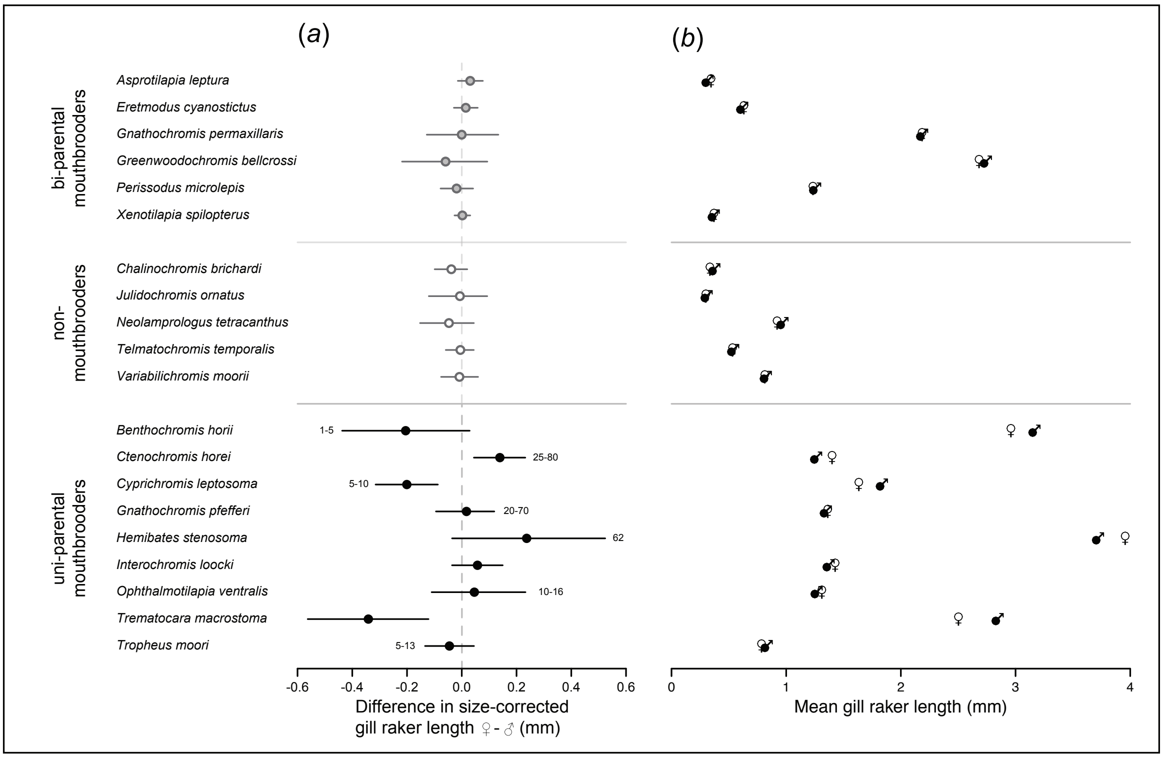

Figure S2: Sexual dimorphism in gill raker lengths. ***(a)*** Mean gill raker length for either sex of each species, illustrating the extent and the direction of sexual dimorphism with respect to the actual gill raker length. The realized trait value in females (mouthbrooding sex) in respect to males does not show a shift in trait values towards a certain gill raker length across all species (optimum), suggesting more than one optimum for mouthbrooding. ***(b)*** Difference in size-corrected gill raker length for each species. Numbers next to the data points indicate clutch size [1].

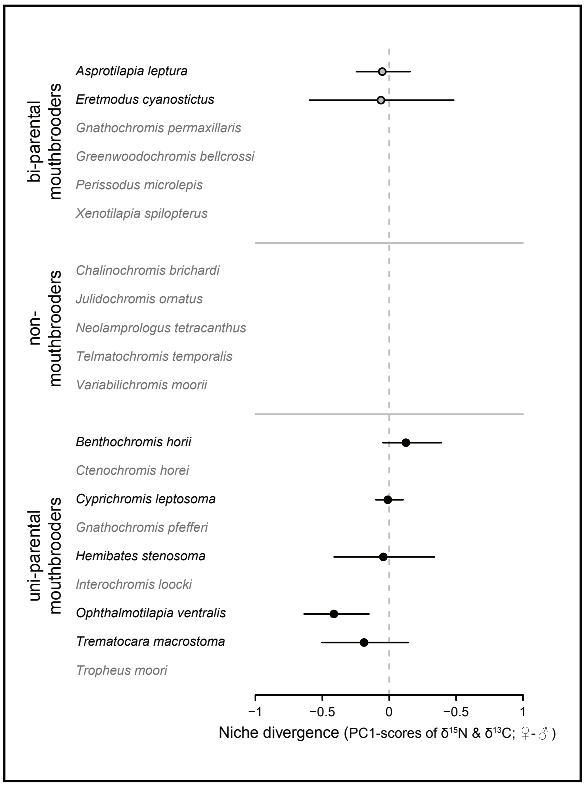

Figure S3: Niche divergence between males and females: Difference in PC1-scores ($\delta^{15}N$ and $\delta^{13}C$ stable isotope signatures) between the sexes showed no evidence for niche divergence between males and females in uni-parental mouthbrooders that are sexually dimorphic in gill raker morphology. Due to missing sex information in the stable isotope data only a subset of species was tested with a very reduced sample size per species (see supplementary table S1). Note that in *Ophthalmotilapia ventrails*, the only species with divergent niche use, males and females differ in habitat preference: While males are territorial in the shallows, females school in deeper waters. Therefore, it is not surprising that the sexes differ in their stable isotope signatures (especially in $\delta^{13}C$).

Table S1. Overview of all 65 Tanganyika cichlid species investigated in this study, including information on taxonomy (species and tribes), breeding mode, and sample sizes. Number of gill raker measurements taken from Muschick et al. [2] are given in brackets.

species information				gill raker measurements					stable isotope analysis			comments	food type (data from [1])
						N_{total}							
species abb.	full name	tribe	breeding mode	N_{males}	$N_{females}$	newly aquired for this study	taken from [2]	total	N_{males}	$N_{females}$	N_{total}		
Altcal	*Altolamprologus calvus*	Lamprologini	non-mouthbrooder (substrate brooder)	0	1	0	(+1)	1	3	NA	10		
Altcom	*Altolamprologus compressiceps*	Lamprologini	non-mouthbrooder (substrate brooder)	2	1	3	(+11)	14	NA	1	10		
Asplep	*Asprotilapia leptura*	Ectodini	bi-parental mouthbrooder	14	15	27	(+8)	35	4	5	10	tested for sexual dimorphism	aufwuchs
Astbur	*Astatotilapia burtoni*	Haplochromini	uni-parental mouthbrooder (maternal)	6	2	0	(+10)	10	4	3	10		
Auldew	*Aulonocranus dewindti*	Ectodini	uni-parental mouthbrooder (maternal)	1	NA	1	(+11)	12	2	3	10		
Benhor	*Benthochromis horii*	Benthochromini	uni-parental mouthbrooder (maternal)	14	7	19	(+3)	22	6	4	10	tested for sex dimorphism	zooplankton
Calmac	*Callochromis macrops*	Ectodini	uni-parental mouthbrooder (maternal)	1	NA	0	(+10)	10	5	2	10		
Chabri	*Chalinochromis brichardi*	Lamprologini	non-mouthbrooder (substrate brooder)	5	5	9	(+4)	13	3	1	10	tested for sex dimorphism	picks sponges & inverebrates
Cphgib	*Cyphotilapia gibberosa*	Cyphotilapiini	uni-parental mouthbrooder (maternal)	2	4	0	(+8)	8	4	1	10		
Ctehor	*Ctenochromis horei*	Tropheini	uni-parental mouthbrooder (maternal)	16	16	30	(+10)	40	6	NA	10	tested for sex dimorphism	picks tiny shrimps & sift worms from sand
Cyafur	*Cyathopharynx furcifer*	Ectodini	uni-parental mouthbrooder (maternal)	4	NA	0	(+9)	9	6	4	10		
Cypcol	*Cyprichromis coloratus*	Cyprichromini	uni-parental mouthbrooder (maternal)	2	1	3		3	5	2	10		
Cyplep	*Cyprichromis leptosoma*	Cyprichromini	uni-parental mouthbrooder (maternal)	17	15	31	(+11)	42	5	4	10	tested for sexual dimorphism	zooplankton
Enamel	*Enantiopus melanogenys*	Ectodini	uni-parental mouthbrooder (maternal)	1	5	0	(+7)	7	9	1	10		
Erecya	*Eretmodus cyanostictus*	Eretmodini	bi-parental mouthbrooder	15	15	30	(+9)	39	4	6	10	tested for sexual dimorphism	filamentous algae
Gnaper	*Gnathochromis permaxillaris*	Limnochromini	bi-parental mouthbrooder	19	16	35		35	NA	NA	10	tested for sexual dimorphism	sucks tiny invertebras from muddy bottom
Gnapfe	*Gnathochromis pfefferi*	Tropheini	uni-parental mouthbrooder (maternal)	7	12	18	(+8)	26	2	2	10	tested for sexual dimorphism	picks shrimps form the substrate
Gralem	*Grammatotria lemairii*	Ectodini	uni-parental mouthbrooder (maternal)	NA	1	0	(+4)	4	3	2	10		
Gwcabe	*Greenwoodochromis abeelei*	Limnochromini	bi-parental mouthbrooder	2	6	8		8	4	1	10		
Gwcbel	*Greenwoodochromis bellcrossi*	Limnochromini	bi-parental mouthbrooder	15	7	22		22	1	1	10	tested for sexual dimorphism	small fish or shrimps (speculative)
Hapmic	*Haplotaxodon microlepis*	Perissodini	bi-parental mouthbrooder	1	2	0	(+15)	15	7	2	10		
Hemste	*Hemibates stenosoma*	Bathybatini	uni-parental mouthbrooder (maternal)	13	11	25		25	6	4	10	tested for sexual dimorphism	small fish
Intloo	*Interochromis loocki*	Tropheini	uni-parental mouthbrooder (maternal)	17	15	31	(+10)	41	1	2	10	tested for sexual dimorphism	diatoms & cyanobacteria
Julorn	*Julidochromis ornatus*	Lamprologini	non-mouthbrooder (substrate brooder)	5	3	7	(+8)	15	NA	NA	10	tested for sexual dimorphism	picks sponges & inverebrates
Lamcal	*Lamprologus callipterus*	Lamprologini	non-mouthbrooder (substrate brooder)	5	NA	3	(+12)	15	1	4	10		
Lamlem	*Lamprologus lemairii*	Lamprologini	non-mouthbrooder (substrate brooder)	1	NA	1	(+5)	6	3	2	10		
Lepatt	*Lepidiolamprologus attenuatus*	Lamprologini	non-mouthbrooder (substrate brooder)	NA	NA	0	(+10)	10	4	3	10		
Lepelo	*Lepidiolamprologus elongatus*	Lamprologini	non-mouthbrooder (substrate brooder)	NA	1	0	(+10)	10	1	1	10		
Leppro	*Lepidiolamprologus profundicola*	Lamprologini	non-mouthbrooder (substrate brooder)	2	2	0	(+5)	5	3	6	10		
Limdar	*Limnotilapia dardennii*	Tropheini	uni-parental mouthbrooder (maternal)	NA	2	2	(+8)	10	3	5	10		
Loblab	*Lobochilotes labiatus*	Tropheini	uni-parental mouthbrooder (maternal)	NA	3	0	(+15)	15	1	2	10		
Neocau	*Neolamprologus caudopunctatus*	Lamprologini	non-mouthbrooder (substrate brooder)	1	5	5	(+10)	15	2	4	10		
Neofas	*Neolamprologus fasciatus*	Lamprologini	non-mouthbrooder (substrate brooder)	5	1	5	(+10)	15	5	NA	10		
Neofur	*Neolamprologus furcifer*	Lamprologini	non-mouthbrooder (substrate brooder)	NA	NA	0	(+1)	1	4	3	10		
Neomod	*Neolamprologus modestus*	Lamprologini	non-mouthbrooder (substrate brooder)	3	NA	3	(+9)	12	6	1	10		
Neomon	*Neolamprologus mondabu*	Lamprologini	non-mouthbrooder (substrate brooder)	NA	NA	0	(+4)	4	5	5	10		
Neomux	*Neolamprologus mustax*	Lamprologini	non-mouthbrooder (substrate brooder)	NA	NA	0	(+2)	2	4	3	10		
Neopro	*Neolamprologus prochilus*	Lamprologini	non-mouthbrooder (substrate brooder)	NA	NA	0	(+1)	1	4	4	10		
Neopul	*Neolamprologus pulcher*	Lamprologini	non-mouthbrooder (substrate brooder)	2	NA	2	(+11)	13	NA	NA	10		
Neosav	*Neolamprologus savoryi*	Lamprologini	non-mouthbrooder (substrate brooder)	2	NA	1	(+11)	12	3	NA	10		
Neosex	*Neolamprologus sexfasciatus*	Lamprologini	non-mouthbrooder (substrate brooder)	NA	2	0	(+8)	8	5	2	10		
Neotet	*Neolamprologus tetracanthus*	Lamprologini	non-mouthbrooder (substrate brooder)	5	4	8	(+6)	14	2	NA	10	tested for sexual dimorphism	snails
Ophnas	*Ophthalmotilapia nasuta*	Ectodini	uni-parental mouthbrooder (maternal)	NA	2	0	(+5)	5	5	5	10		
Ophven	*Ophthalmotilapia ventralis*	Ectodini	uni-parental mouthbrooder (maternal)	16	12	27	(+11)	38	5	5	10	tested for sexual dimorphism	phytoplankton & aufwuchs
Pcybri	*Paracyprichromis brieni*	Cyprichromini	uni-parental mouthbrooder (maternal)	1	NA	1	(+5)	6	4	4	10		
Permic	*Perissodus microlepis*	Perissodini	bi-parental mouthbrooder	15	21	30	(+10)	40	1	1	10	tested for sexual dimorphism	fish scales
Peteph	*Petrochromis ephippium*	Tropheini	uni-parental mouthbrooder (maternal)	NA	1	0	(+5)	5	NA	NA	10		
Petfam	*Petrochromis famula*	Tropheini	uni-parental mouthbrooder (maternal)	NA	3	0	(+10)	10	3	NA	10		
Petfas	*Petrochromis fasciolatus*	Tropheini	uni-parental mouthbrooder (maternal)	1	0	1		1	3	3	10		
Petmac	*Petrochromis macrognathus*	Tropheini	uni-parental mouthbrooder (maternal)	NA	2	0	(+10)	10	6	3	10		
Petpol	*Petrochromis polyodon*	Tropheini	uni-parental mouthbrooder (maternal)	2	NA	0	(+7)	7	2	4	10		
Plestr	*Plecodus straeleni*	Perissodini	bi-parental mouthbrooder	NA	2	0	(+10)	10	6	2	10		
Psccur	*Pseudosimochromis curvifrons*	Tropheini	uni-parental mouthbrooder (maternal)	3	2	0	(+10)	10	5	4	10		
Simdia	*Simochromis diagramma*	Tropheini	uni-parental mouthbrooder (maternal)	NA	2	0	(+10)	10	1	5	10		
Teldho	*Telmatochromis dhonti*	Lamprologini	non-mouthbrooder (substrate brooder)	3	0	3		3	NA	NA	10		
Teltem	*Telmatochromis temporalis*	Lamprologini	non-mouthbrooder (substrate brooder)	5	4	9	(+4)	13	6	NA	10	tested for sexual dimorphism	filamentous algea, plankton
Tremac	*Trematocara macrostoma*	Trematocarini	uni-parental mouthbrooder (maternal)	8	7	15		15	6	4	10	tested for sexual dimorphism	fish or zooplankton (speculative)
Tremar	*Trematocara marginatum*	Trematocarini	uni-parental mouthbrooder (maternal)	0	1	1		1	7	5	12		
Trenig	*Trematocara nigrifrons*	Trematocarini	uni-parental mouthbrooder (maternal)	0	12	12		12	5	15	20		
Treuni	*Trematocara unimaculatum*	Trematocarini	uni-parental mouthbrooder (maternal)	7	3	10		10	4	2	9		
Tromoo	*Tropheus moorii*	Tropheini	uni-parental mouthbrooder (maternal)	15	16	30	(+10)	40	2	NA	10	tested for sexual dimorphism	filamentous algea
Tylpol	*Tylochromis polylepis*	Tylochromini	uni-parental mouthbrooder (maternal)	NA	NA	0	(+3)	3	6	2	10		
Varmoo	*Variabilichromis moorii*	Lamprologini	non-mouthbrooder (substrate brooder)	6	9	9	(+10)	19	NA	NA	10	tested for sexual dimorphism	filamentous algea, diatoms, ostracods
Xenfla	*Xenotilapia flavipinnis*	Ectodini	bi-parental mouthbrooder	1	1	0	(+7)	7	NA	NA	10		
Xenspi	*Xenotilapia spilopterus*	Ectodini	bi-parental mouthbrooder	17	15	31	(+5)	36	1	1	10	tested for sexual dimorphism	insect larvea, rarely zooplankton
Total	***65 species***	***13 tribes***	***3 breeding modes***	***305***	***295***	***508***	***(+427)***	***935***	***224***	***161***	***661***	***20 species***	

Table S2: Summary tables of tests for sexual dimorphism in 20 cichlid species, and for the association between sexual dimorphism and breeding mode. Statistically significant p-values ($p < 0.05$) are highlighted in bold. ***(a)*** Testing for a difference in mean size-corrected gill raker length between females and males within each species. ***(b)*** Testing mean dimorphism per breeding mode for deviation from zero. ***(c)*** ANOVA statistics on mean absolute dimorphism among the breeding modes. ***(d)*** Pairwise comparisons of absolute difference in mean sexual dimorphism in gill raker length among breeding modes.

(*a*)

breeding mode	species	difference f-m	CI_{min}	CI_{max}	*p*-value
bi-parental mouthbrooders	*Asprotilapia leptura*	0.031	-0.014	0.077	0.208
	Eretmodus cyanostictus	0.015	-0.029	0.058	0.535
	Gnathochromis permaxillaris	0.000	-0.128	0.134	0.998
	Greenwoodochromis bellcrossi	-0.059	-0.218	0.093	0.476
	Perissodus microlepis	-0.019	-0.077	0.041	0.571
	Xenotilapia spilopterus	0.002	-0.027	0.031	0.895
non-mouthbrooders	*Chalinochromis brichardi*	-0.038	-0.099	0.020	0.276
	Julidochromis ornatus	-0.007	-0.120	0.093	0.889
	Neolamprologus tetracanthus	-0.047	-0.152	0.044	0.391
	Telmatochromis temporalis	-0.005	-0.059	0.044	0.853
	Variabilichromis moorii	-0.009	-0.076	0.060	0.828
uni-parental mouthbrooders	*Benthochromis horii*	-0.205	-0.437	0.029	0.126
	Ctenochromis horei	0.139	0.044	0.232	***0.006***
	Cyprichromis leptosoma	-0.201	-0.315	-0.087	***0.003***
	Gnathochromis pfefferi	0.017	-0.094	0.119	0.758
	Hemibates stenosoma	0.237	-0.035	0.524	0.111
	Interochromis loocki	0.057	-0.036	0.150	0.252
	Ophthalmotilapia ventralis	0.046	-0.110	0.233	0.611
	Trematocara macrostoma	-0.341	-0.563	-0.122	***0.014***
	Tropheus moori	-0.045	-0.134	0.045	0.34

(*b*)

breeding mode	$mean_{mode}$	CI_{min}	CI_{max}	*p*-value
bi-parental mouthbrooders	-0.005	-0.029	0.015	0.722
non-mouthbrooders	-0.021	-0.037	-0.006	0.068
uni-parental mouthbrooders	-0.033	-0.152	0.079	0.617

(*c*)

model	F-statistics	*p*-value lm()	*p-value phylANOVA()*
abs(dimorphism) ~ mode	F = 6.19	***0.007***	**0.17**

(*d*)

comparison	mean difference	*p*-value lm()	*p-value phylANOVA()*
difference abs(**UNI**) *vs.* abs(**BI**)	0.122	***0.015***	***0.031***
difference abs(**UNI**) *vs.* abs(**NON**)	0.122	***0.022***	0.172
difference abs(**NON**) *vs.* abs(**BI**)	<0.001	0.995	1.000

Table S3: Summary of the break-point model fitted to investigate the association between sexual dimorphism in gill raker length with trophic ecology within uni-parental mouthbrooders. Statistically significant p-values (p < 0.05) are highlighted in bold.

model	lm()				davies.test()	segmented.lm()			phylANOVA()	
	R^2	adjusted R^2	F-statistics	*p* -value	*p* -value	R^2	breakpoint	adjusted R^2	t-statistics	*p* -value
$dimorph_{UNI}$ ~ $PC1_{UNI}$	0.56	0.50	8.92	***0.020***	***0.044***	0.87	0.344	0.796	-	-
$dimorph_{UNI(PC1<0.34)}$ *vs.* $dimorph_{UNI(PC1>0.34)}$	-	-	-	-	-	-	-	-	4.8	***0.001***

References

1. Konings A. 2015 Tanganyika Cichlids in their natural habitat. 3th Editio. El Paso: Cichlid Press.
2. Muschick M, Nosil P, Roesti M, Dittmann MT, Harmon L, Salzburger W. 2014 Testing the stages model in the adaptive radiation of cichlid fishes in East African Lake Tanganyika. Proc. R. Soc. B Biol. Sci. 281, 20140605–20140605. (doi:10.1098/rspb.2014.0605)
